# Salivary Gland Extract Modulates the Infection of Two *Leishmania enriettii* Strains by Interfering With Macrophage Differentiation in the Model of *Cavia porcellus*

**DOI:** 10.3389/fmicb.2018.00969

**Published:** 2018-05-29

**Authors:** Lucélia J. Pinheiro, Larissa F. Paranaíba, Adriano F. Alves, Patrícia M. Parreiras, Nelder F. Gontijo, Rodrigo P. Soares, Wagner L. Tafuri

**Affiliations:** ^1^Departamento de Patologia Geral, Instituto de Ciências Biológicas, Universidade Federal de Minas Gerais, Belo Horizonte, Brazil; ^2^Departamento de Parasitologia, Instituto de Ciências Biológicas, Universidade Federal de Minas Gerais, Belo Horizonte, Brazil; ^3^Instituto René Rachou, Fundação Oswaldo Cruz Belo Horizonte, Brazil

**Keywords:** *Leishmania Mundinia enriettii*, *Cavia porcellus*, salivary gland extract, immunopathology, CD163+/L1+ macrophages

## Abstract

The subgenus *Mundinia* includes several *Leishmania* species that have human and veterinary importance. One of those members, *Leishmania Mundinia enriettii* was isolated from the guinea pig *Cavia porcellus* in the 1940s. Several histopathological studies have already been performed in this species in the absence of salivary gland extract (SGE), which are determinant and the early and future events of the infection. Our main hypothesis is that SGE could differentially modulate the course of the lesion and macrophage differentiation caused by avirulent and virulent *L. enriettii* strains. Here, the *C. porcellus* nasal region was infected using needles with two strains of *L. enriettii* (L88 and Cobaia) in the presence/absence of SGE and followed for 12 weeks. Those strains vary in terms of virulence, and their histopathological development was characterized. Some L88-infected animals could develop ulcerated/nodular lesions, whereas Cobaia strain developed non-ulcerated nodular lesions. Animals experimentally inoculated developed a protuberance and/or lesion after the 4th and 5th weeks of infection. Macroscopically, the size of lesion in L88-infected animals was smaller in the presence of SGE. Remarkable differences were detected microscopically in the presence of SGE for both strains. After the 6th and 7th weeks, L88-infected animals were heavily parasitized with an intense inflammatory profile bearing amastigotes and pro-inflammatory cells compared to those infected by Cobaia strain. Morphometry analysis revealed that L1+ macrophages were abundant in the L88 infection, but not in the Cobaia infection. In the presence of SGE, an increased CD163+ macrophage infiltrate by both strains was detected. Interestingly, this effect was more pronounced in Cobaia-infected animals. This study showed the role of SGE during the course of *L. enriettii* (strains L88 and Cobaia) infection and its role in modulating macrophage attraction to the lesion site. SGE decreased L1+ macrophages and this may favor an escaping mechanism for L88 parasites. On the other hand, in the presence of SGE, an increase in CD163+ cells during Cobaia infection may be important for its control. Although both strains healed at the end of the infection, the role of SGE was determinant for the kinetics of the immunopathological events in this dermotropic species.

## Introduction

Parasites previously classified into the “*Leishmania enriettii* complex” (Paranaiba et al., 2017a) were recently renamed in the subgenus *Mundinia*: *Leishmania* (*Mundinia*) *enriettii*, *Leishmania* (*Mundinia*) *martiniquensis, Leishmania* (*Mundinia*) *siamensis*, and *Leishmania* (*Mundinia*) *macropodum* (Espinosa et al., 2016; Paranaiba et al., 2017b). The Subgenus *Mundinia* has emerged a great interest in the field of parasitology, for presenting pathogenic agents that causes visceral leishmaniasis (VL) and cutaneous leishmaniasis (CL) in humans and other vertebrates.

Besides its medical importance, *Mundinia* parasites are also able to infect animals denoting high phenotypic plasticity. They were isolated from guinea pigs, kangaroos, horses, and bovines in different parts of the world. The first description of *L. enriettii* occurred in Brazil in 1945 (Muniz and Medina, 1948) and later in 1994 (Machado et al., 1994), when those parasites were isolated from guinea pigs (*Cavia porcellus*). Later, a similar isolate was found in Australia in the red kangaroo *Macropus rufus*, and this finding called the attention to this at that time so called “*L. enriettii* complex”. Nowadays, the Australian isolate is named *Leishmania macropodum* (Espinosa et al., 2016). In addition, putative autochthonous cases of “*L. siamensis*” were also isolated from bovines in Switzerland (Lobsiger et al., 2010) and horses in Florida, United States (Reuss et al., 2012). However, in most of those cases a detailed histopathological analysis of the lesions was not fully assessed. In the red kangaroo, where *L. macropodum* is considered an opportunistic agent, preliminary observations showed that their nodular CL lesions presented moderate to marked infiltration with macrophages containing amastigotes (Dougall et al., 2009). However, a follow-up-controlled study on histopathology in *Mundinia* parasites is not available yet.

The most studied species in terms of histopathology in the *Mundinia* subgenus is *L. enriettii*. This species has been used as an *in vivo* model for CL in several publications (Paraense, 1953; Bryceson et al., 1974; Belehu and Turk, 1976; Schottelius, 1987). In general, the histopathological description of dermotropic leishmaniasis is represented by a chronic inflammatory reaction exhibiting a dense mononuclear exudate with the presence/absence of necrosis. This is often observed for dermotropic species including *Leishmania guyanensis*, *Leishmania braziliensis*, *Leishmania amazonensis*, and *Leishmania shawi*. However, in *L. amazonensis*, the exudate is characterized by vacuolized macrophages intensely parasitized by amastigotes with rare lymphocytes named Virchow granulomas (de Magalhães et al., 1986; Silveira et al., 2004; Marques et al., 2017). Similarly, CL lesions caused by *L. enriettii* are also characterized by a chronic inflammatory infiltrate of mononuclear cells. In the dermis, macrophages are usually densely parasitized by intracellular amastigote forms (Muniz and Medina, 1948; Paraense, 1953; Bryceson et al., 1974). An unknown aspect in the histopathology of leishmaniasis is what type of macrophages is involved in those processes. L1 and CD163 macrophages are often present in inflammatory process in several diseases especially during control and repair mechanisms (Van den Heuvel et al., 1999; Steinbrink et al., 2000; Li et al., 2015). Since the lesions of *L. enriettii* often heal after 8–11 weeks (Paranaíba et al., 2015), the role of those cells is still unknown in this process. Besides, those histopathological studies with *L. enriettii* have not used the salivary gland extract (SGE) together with parasite inoculum. Our main goal is to ascertain the role of SGE in modulating the course of infection and macrophage differentiation caused by *L. enriettii* strains exhibiting different degrees of virulence.

Several factors are released by the sand fly in the bite site (Gomes et al., 2012). Besides saliva, those include proteophosphoglycans (PPGs) and exosomes (Rogers, 2012; Diniz Atayde et al., 2015). Sand fly saliva has a large number of immunomodulatory substances that are important during the host–parasite interaction (Ribeiro and Francischetti, 2003). In the case of *Lutzomyia longipalpis*, the main vector of VL in Latin America, the most important vasodilator is maxadilan (revised by Soares and Turco, 2003). Besides maxadilan, a wide range of different immunomodulators, known to play a pivotal role during *Leishmania* infection have been reported (Sacks and Kamhawi, 2001). Those molecules may vary according to the sand fly vectors (Volf et al., 2000) and are determinant for the *Leishmania* species they transmit. For example, early studies using SGE exacerbated *Leishmania* infection in mice by *Leishmania major* and *L. amazonensis* (Titus and Ribeiro, 1988; Theodos et al., 1991). Recently, the role of IL-1β was shown to be crucial for *Leishmania donovani* visceralization (Dey et al., 2018). This phenomenon was affected not only by saliva but also by the presence of sand fly microbiota. Consistent with those observations, recently SGE from *L. longipalpis* also modulated the size of the lesion in *C. porcellus* infected with L88 strain of *L. enriettii* (Paranaíba et al., 2015). However, no demonstration of the histopathological events in the presence of SGE in different strains of this species is provided.

Here, we performed a more detailed follow-up histological evaluation using two World Health Reference strains (L88 and Cobaia) with different degrees of virulence. The former causes ulcerated lesions and the latter does not ulcerate but causes nodular lesions. Also, we provide an immunohistochemistry analysis by probing the presence of the parasite and macrophages (L1 and CD163) during the early and later steps of the infection. The proteins L1/Calprotectin and CD163 are expressed only in monocytes and subpopulations of tissue macrophages (Van den Heuvel et al., 1999). The L1 is used to indicate inflammatory processes, originating in the initial period of infection and CD163 is present in different populations of macrophages related to processes of tissue repair. An important feature of this study is to perform all experiments in the presence/absence of SGE, a condition that mimics transmission in nature.

## Materials and Methods

### *Leishmania enriettii* Strains and Cell Culture

World Health Reference strains of *L. (M.) enriettii* (L88 and Cobaia) were grown in M199 medium as previously reported at 25°C until early-stationary phase (Soares et al., 2002). Frozen infective stocks used in our previous publication (Paranaíba et al., 2015) were used for the experimental infections.

### Sand Fly Colony

We have used a sand fly colony of *L. longipalpis* established at the Parasitology Department at Federal University of Minas Gerais. This colony originated from captured females from Teresina, Piaui State, Brazil and were reared as described elsewhere (Modi and Tesh, 1983). This sand fly species can sustain infection by *L. enriettii* (Seblova et al., 2015). Their salivary glands were dissected following the same methodology as previously reported (Paranaíba et al., 2015).

### Ethics Statement and *in Vivo* Experiments

This work was approved by the Internal Ethics Committee in Animal Experimentation (CEUA) of Fundação Oswaldo Cruz (FIOCRUZ) (Protocol p-0297-06). The animals were kept in appropriate cages with free access to water and food in a temperature-controlled room (24°C) with light/dark cycle (12 h light from 7:00 a.m.) at the Animal Facilities at the René Rachou Institute/FIOCRUZ. Nasal skin fragments were used for 78 (72 infected + 6 controls) male guinea pigs, 3 weeks old. Those were divided in four experimental groups including: (1) 18 animals (L88 strain); (2) 18 animals (L88 + SGE); (3) 18 animals (Cobaia strain) and (4) 18 animals (Cobaia strain + SGE). Negative controls included: two animals (salina), two animals (no inoculation), and two animals (SGE only). Animals were inoculated by needle with 0.1 mL of salina containing 1× 10^5^ promastigotes in the presence/absence of SGE from 1/2 gland as previously reported (Paranaíba et al., 2015). Animals were followed for 12 weeks and euthanized (3 per group) every 2 weeks for histological evaluation.

### Histological Evaluation, Immunohistochemistry, and Morphometry

Nasal skin fragments of experimentally infected animals were collected and fixed in 10% buffered formalin solution pH 7.2 for 72 h, as described by Alves et al. (2013). The paraffin blocks containing the skin fragments were cut using a microtome in the thickness of 3–4 μm and mounted on slides. These slides were designed for histological analysis following routine histology techniques (Hematoxylin and Eosin – H & E). Immunohistochemistry (IHC) was performed for parasite detection using canine hyper serum as the probing antibody (Tafuri et al., 2004). For macrophage detection, mAbs L1/calprotectin [Mouse anti Human Anti-L1 (Calprotectin) 1/100 MCA 874G – Serotec] and CD163 (Rb antiCD163 1/100 REF: E 18684 – Spring) were used (Stříž and Trebichavský, 2004; Goto et al., 2008). The histological slides were analyzed under light microscopy and alterations were classified, semi-quantitatively, taking into account the intensity of the affected fragments, being classified as: discrete (<25%), moderate (25–50%) and intense (>50%) (Figueiredo et al., 2010; **Table 1**). All images were visualized using the QuiColor 3 microchamber (Olympus^®^, Pennsylvania, United States) and scanned BX 40 (Olympus^®^) microscope (magnification 40×). For morphometric semi-quantitative analysis, 25 fields of each slide were randomly photographed. Quantification of parasitism and mononuclear cells for both strains of *L. enriettii* were performed in KS300 software. Data were analyzed using GraphPad Prism 5.0. Statistical analyses used Kruskal–Wallis test followed by Dunn’s post-test. *P*-values <0.05 were considered statistically significant.

**Table 1:** Inflammation scoring system.

Inflammation	Score
Absent	0
Discreet	1
Moderate	2
Intense	3
Focal	1
Diffuse	2


## Results

### Macroscopic/Microscopic Evaluation and Morphometry

**FIGURE 1:**
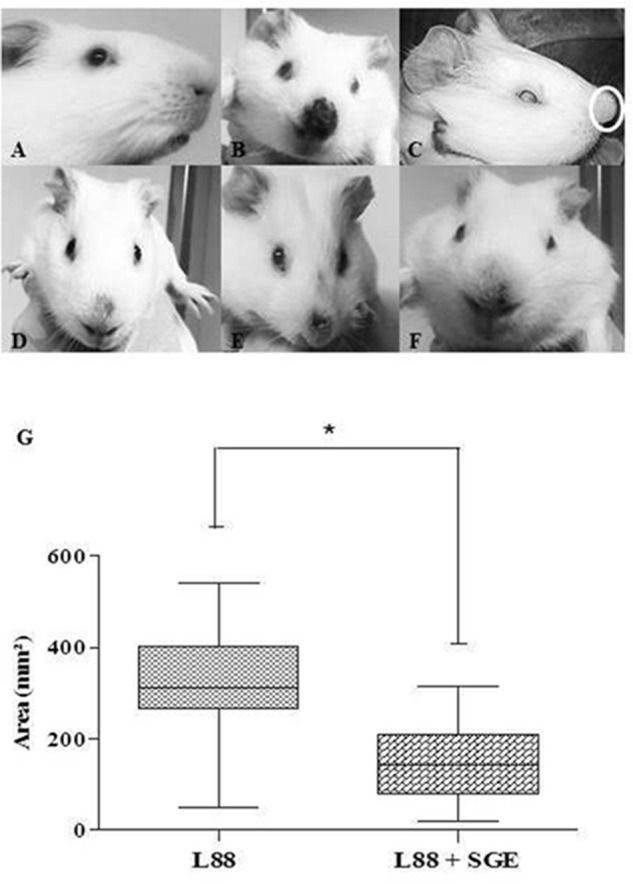
Nasal region of *C. porcellus* infected with *L. enriettii* (L88 and Cobaia) strains in the presence/absence of salivary gland extract (SGE). **(A)** Uninfected *C. porcellus* (negative control). Macroscopical evalution at 6 **(B–E)** and 10 weeks **(F)** PI. **(B)** L88-infected *C. porcellus*. **(C)** Cobaia-infected *C. porcellus* showing a nasal protuberance (nodular lesion – white circle). **(D–F)** Lesion progression towards healing **(F)** in L88-infected *C. porcellus* in the presence of SGE showing alopecia and hyperemia **(D,E)**. **(G)** Impact of SGE in the lesion size (mm^2^) of L88-infected *C. porcellus* throughout the experiment (^∗^ indicates statistical difference, *p* < 0.05).

Under macroscopic analysis, we observed skin lesions in all infected animals (**Figures 1B–F**) except in an uninfected control (**Figure 1A**). As expected, some L88-infected *C. porcellus* developed ulcerative/nodular cutaneous lesions (18/18) in the 5th–6th weeks PI (**Figures 1B,E,F**). However, some L88-infected animals (9/18) did not exhibit ulcerated lesion showing a marked nodule exhibiting hyperemia (**Figure 1D**). Cobaia-infected animals developed a non-ulcerated nodular lesion (**Figure 1C**, white circle) regardless of the presence/absence of SGE. In the presence of SGE, L88 lesions were smaller in size (**Figure 1G**). Lesions started to heal usually 8–10 weeks PI and healing was faster in the group exposed to SGE + parasites.

**FIGURE 2:**
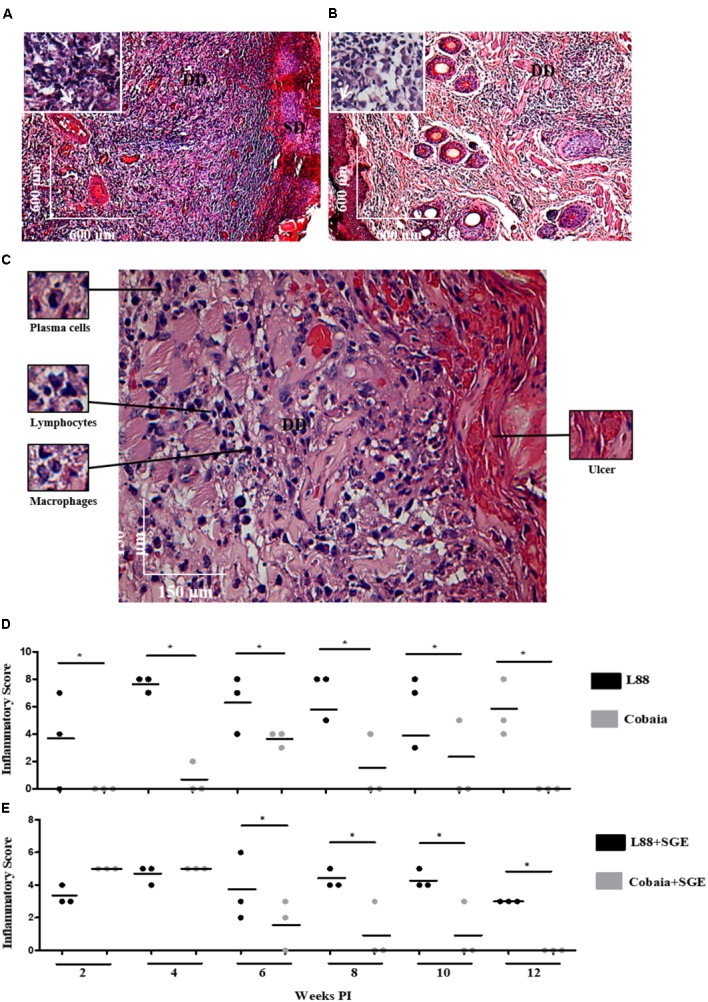
Histopathology (HE) of *C. porcellus* infected with *L. enriettii* (L88 and Cobaia) strains in the absence of salivary gland extract (SGE). **(A)** L88-infected animals exhibited an intense and diffuse chronic inflammatory process. Lower magnification showing a presence of an intense mononuclear exudate throughout the dermis. Epithelial ruptures with crust formation were a consequence of proteinaceous exudate. Amplified section on the left showing amastigote forms of *Leishmania* inside macrophages (arrows). **(B)** Cobaia-infected animals in lower magnification showing a discrete focal chronic inflammatory reaction. Amplified section on the left showing intracellular amastigote forms inside macrophages (arrow). **(C)** Histological section of the nasal region of L88-infected *C. porcellus* in higher magnification showing an intense and diffuse chronic inflammatory reaction. Amplified sections on the left side showing plasma cells, lymphocytes, and macrophages. On the right side, an epithelial rupture with crust formation (ulcer) as a consequence of proteinaceous exudate. Inflammatory Scores of *C. porcellus* infected with *L. enriettii* (L88 and Cobaia) strains in the absence **(D)** and presence **(E)** of SGE at 2, 4, 6, 8, 10 and 12 weeks PI. DD, deep dermis; SD, superficial dermis. Asterisks indicate statistical significance between groups at *p* < 0.05.

Microscopic analysis confirmed previous observations for dermotropic *Leishmania* species (Alves et al., 2013; Marques et al., 2017). L88-infected animals displayed an intense and diffuse chronic inflammatory process. The cellular exudate composed of mononuclear cells (plasma cells, lymphocytes, and macrophages) was diffuse throughout the dermis. Epithelial ruptures with crust formation were a consequence of proteinaceous exudate (**Figures 2A–C**). Macrophages located in the superficial or deep dermis were highly parasitized with amastigotes (**Figures 2A,B**, amplified sections). Some visible features of the macrophage included a vacuolated cytoplasm with a low stained vesicular nucleus (**Figure 2C**, amplified). In the absence of SGE, morphometric semi-quantitative analysis showed that L88 strain exhibited larger inflammatory areas in each week compared to Cobaia strain (**Figure 2D**). Those features were mainly observed between the 4th and 10th weeks of infection for L88, whereas for Cobaia strains those peaked at the 6th week PI. Different from what was observed in the absence of SGE, in its presence the two strains behave similarly in the second and fourth weeks PI. After the 6th week the higher proinflammatory profile of L88 strain persists until the end of the experiment. It is interesting to notice that even after healing of the ulcers in the L88 strain that internally there was still an inflammatory infiltrate (**Figures 2D,E**).

**FIGURE 3:**
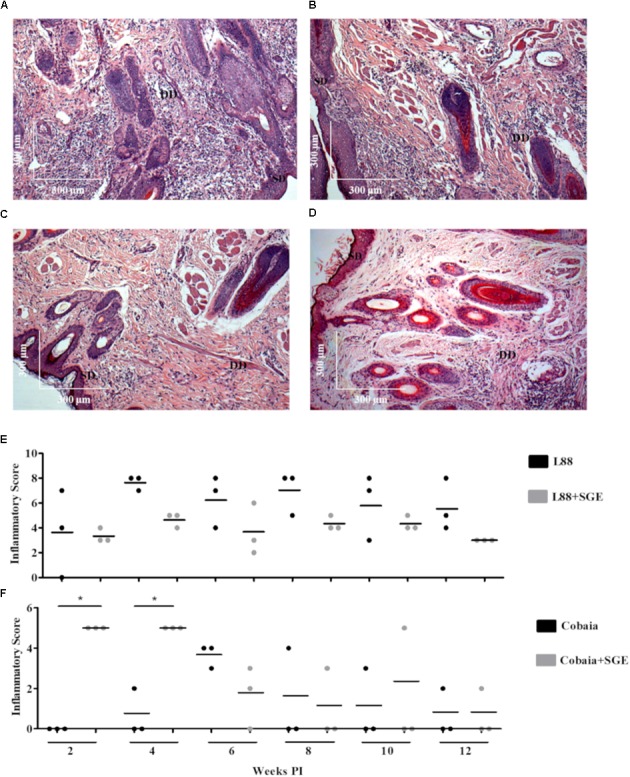
Histopathology (HE) of *C. porcellus* infected with *L. enriettii* (L88 and Cobaia) strains in the absence/presence of salivary gland extract (SGE). L88-infected animals exhibited an intense and diffuse chronic inflammatory process at 6 weeks PI in the absence **(A)** and presence **(B)** of SGE. In the absence of SGE Cobaia-infected animals exhibited a lower chronic inflammatory reaction **(C)** than in the presence of SGE **(D)** at 4 weeks PI. Inflammatory Scores of *C. porcellus* infected with *L. enriettii* strains L88 **(E)** and Cobaia **(F)** in the absence/presence of SGE at 2, 4, 6, 8, 10 and 12 weeks PI. DD deep dermis, SD superficial dermis. Asterisks indicate statistical significance between groups at *p* < 0.05.

Depending on the strain, the presence of SGE modulated the inflammatory infiltrate during the course of infection (**Figures 3A–D**). In L88-infected animals there was no difference in the inflammatory infiltrated during the course of the infection (**Figures 3A,B,E**). Different from L88, in the Cobaia strain, the presence of SGE was determinant especially during the first month PI (**Figures 3C,D,F**). Those data were confirmed after morphometry, where the presence of SGE was responsible for an increase in the inflammatory infiltrate (**Figure 3F**). As expected, negative controls represented by saline, SGE and uninfected animals did not show any alterations (Supplementary Figures S1A–C).

### Immunohistochemistry (IHC)

**FIGURE 4:**
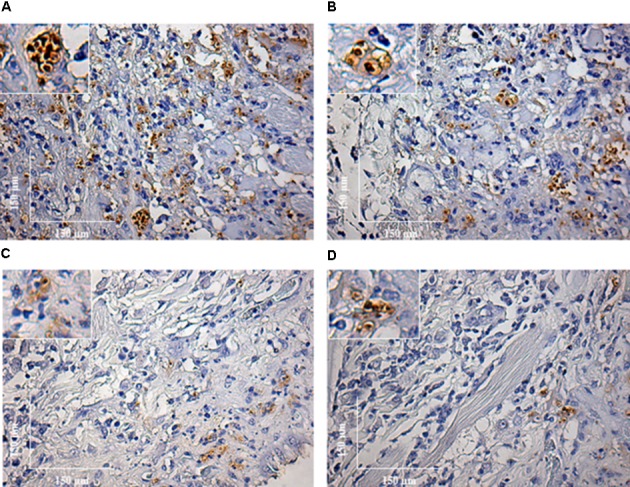
Immunohistochemistry (IHC) of *C. porcellus* infected with *L. enriettii* (L88 and Cobaia) strains in the absence/presence of salivary gland extract (SGE). L88-infected animals exhibited an intense amastigote staining at 6 weeks PI in the absence **(A)** and presence **(B)** of SGE. Cobaia-infected animals exhibited a lower amastigote staining not only in the absence **(C)** but also in the presence of SGE **(D)** at 6 weeks PI. Amplified areas in the upper left side showing amastigotes inside macrophages.

**FIGURE 5:**
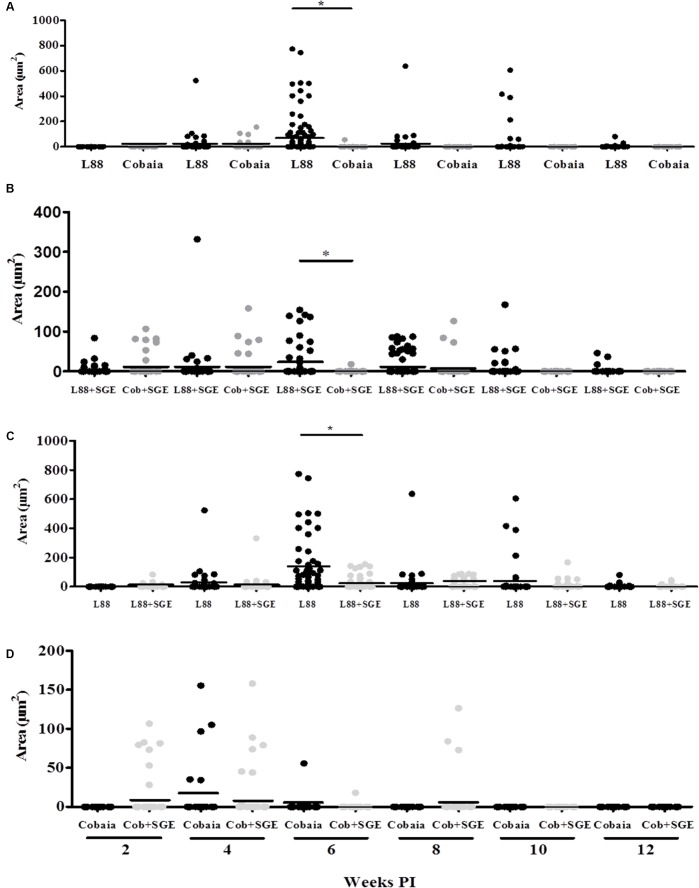
Morphometric analysis (μm^2^) of the immunohistochemistry (IHC) of *C. porcellus* infected with *L. enriettii* (L88 and Cobaia) strains in the absence/presence of salivary gland extract (SGE). Areas of amastigote staining in L88 versus Cobaia strains in the absence **(A)** or presence **(B)** of SGE. L88 **(C)** and Cobaia **(D)** strains in the absence/presence of SGE at 2, 4, 6, 8, 10 and 12 weeks PI. Asterisks indicate statistical significance between groups at *p* < 0.05.

During the HE preparations, the visualization of the parasites is sometimes difficult depending of the strain and parasite load. For this reason, imunocitochemistry (IHC) was employed to quantify the immunolabeled amastigotes. This technique successfully detected amastigotes from both strains of *L. enriettii* using the canine hyper serum as the probing antibody. Those were easily visible by brownish staining without background. Similar to HE, a higher parasite load was detected in all animals infected with L88 strain in the presence/absence of SGE during the 6th week PI compared to Cobaia strain (**Figures 4A–D**). Those observations were confirmed by morphometry, where IHC was more sensitive than HE in detecting SGE influence for L88 strain, but not Cobaia strain, especially during the 6th week PI (**Figures 5A–D**).

**FIGURE 6:**
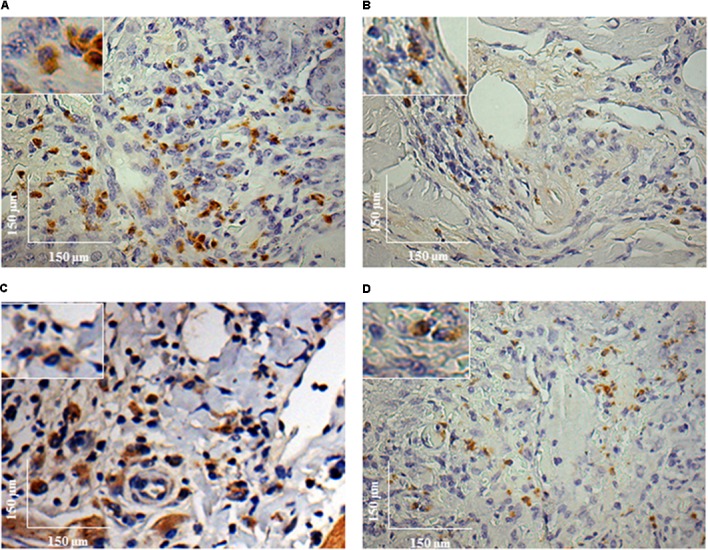
Immunohistochemistry (IHC) using anti-L1 mAb of *C. porcellus* infected with *L. enriettii* (L88 and Cobaia) strains in the absence/presence of salivary gland extract (SGE). L88-infected animals exhibited an intense L1 positive cells staining at 8 weeks PI in the absence **(A)** and presence **(B)** of SGE. In the absence of SGE, Cobaia-infected animals exhibited a higher L1 positive cells staining **(C)** than in the presence of SGE **(D)** at 12 weeks PI. Amplified areas in the upper left side showing macrophage staining.

**FIGURE 7:**
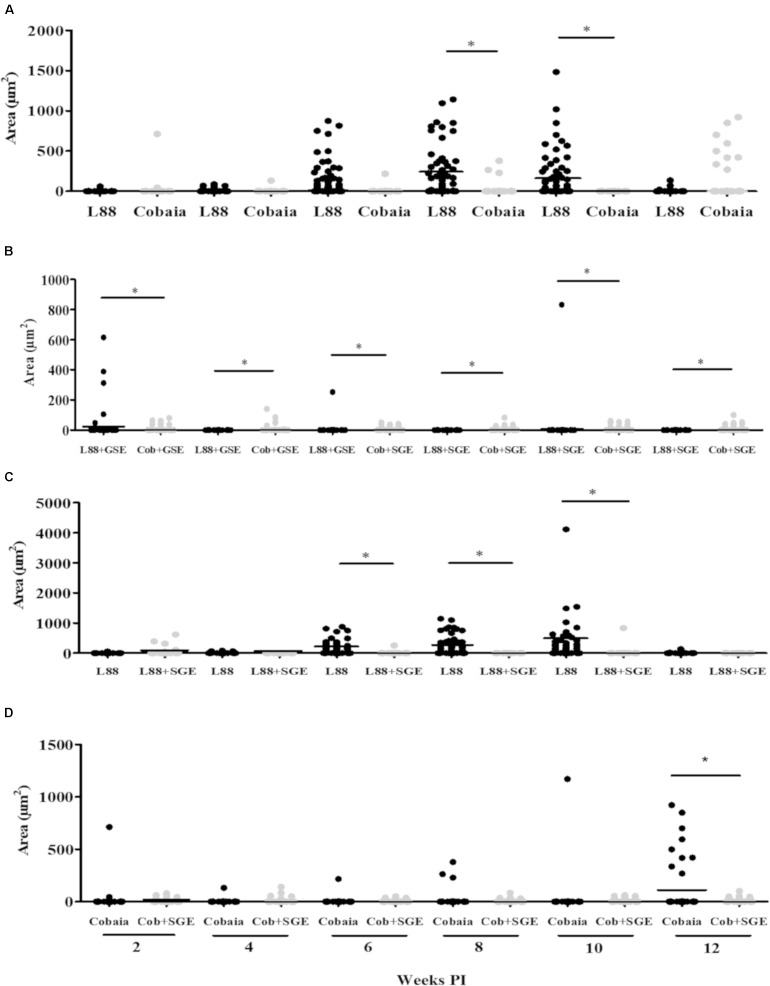
Morphometric analysis (μm^2^) of the immunohistochemistry (IHC) of *C. porcellus* infected with *L. enriettii* (L88 and Cobaia) strains in the absence/presence of salivary gland extract (SGE). Areas of L1 positive cells staining in L88 versus Cobaia strains in the absence **(A)** or presence **(B)** of SGE. L88 **(C)** and Cobaia **(D)** strains in the absence/presence of SGE at 2, 4, 6, 8, 10 and 12 weeks PI. Asterisks indicate statistical significance between groups at *p* < 0.05.

**FIGURE 8:**
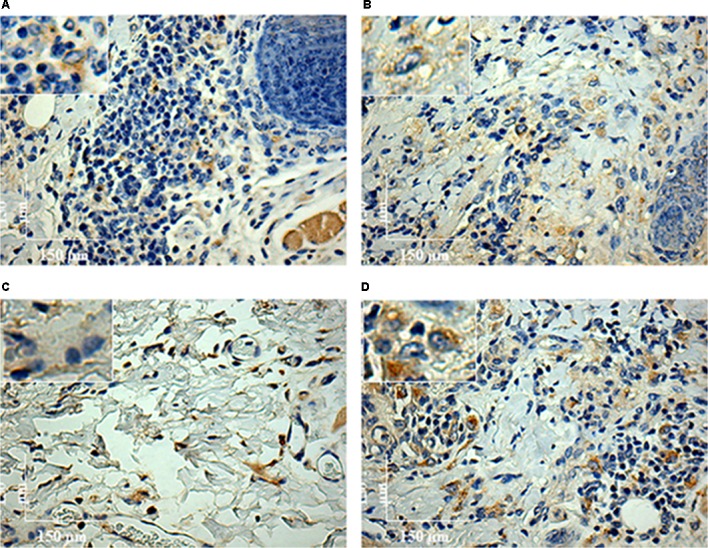
Immunohistochemistry (IHC) using anti-CD163 mAb of *C. porcellus* infected with *L. enriettii* (L88 and Cobaia) strains in the absence/presence of salivary gland extract (SGE). In the absence of SGE, L88-infected animals exhibited an intense CD163 positive cells staining **(A)** than in the presence **(B)** of SGE at 4 weeks PI. In the absence of SGE, Cobaia-infected animals exhibited a lower CD163 positive cells staining **(C)** than in the presence of SGE **(D)** at 6 weeks PI. Amplified areas in the upper left side showing macrophage staining.

**FIGURE 9:**
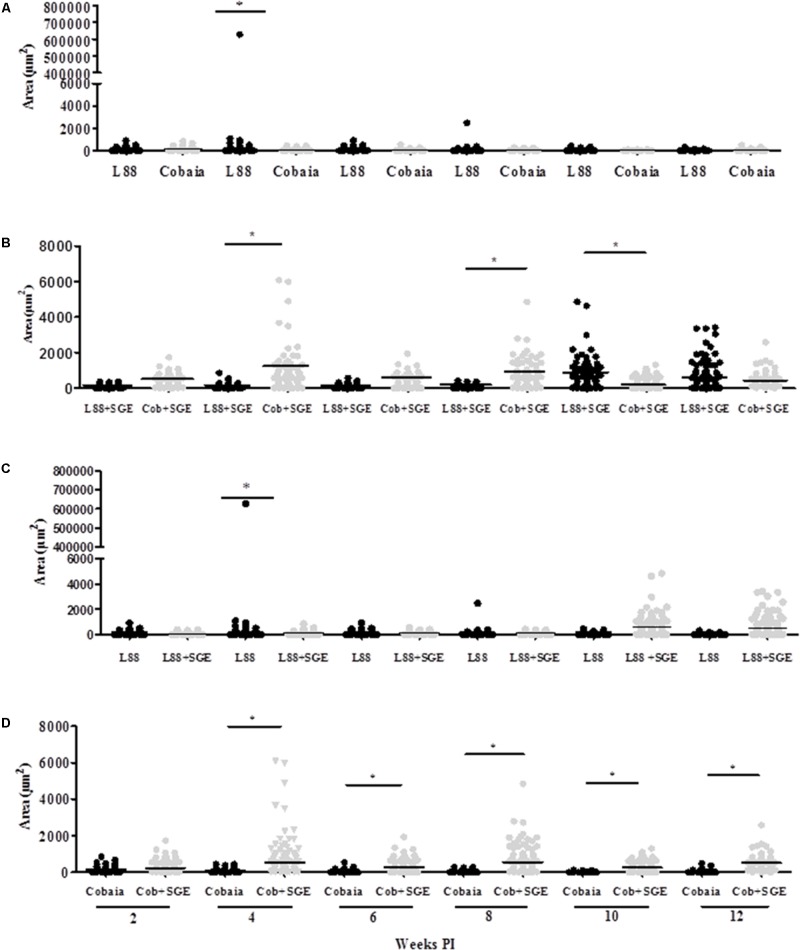
Morphometric analysis (μm^2^) of the immunohistochemistry (IHC) of *C. porcellus* infected with *L. enriettii* (L88 and Cobaia) strains in the absence/presence of salivary gland extract (SGE). Areas of CD163 positive cells staining in L88 versus Cobaia strains in the absence **(A)** or presence **(B)** of SGE. L88 **(C)** and Cobaia **(D)** strains in the absence/presence of SGE at 2, 4, 6, 8, 10 and 12 weeks PI. Asterisks indicate statistical significance between groups at *p* < 0.05.

To characterize the inflammatory macrophages on infected tissues, mAbs anti-L1 (Calprotectin) and anti-CD163 were used. Control animals inoculated with saline and/or SGE alone did not show any positive immuno-labeled cells (Supplementary Figures S1D,E). Only infected animals showed L1 and CD163 positive cells in skin tissues (**Figure 6**). In general, L1 cells were diffusely distributed in the superficial dermis or in focus around blood vessels and skin glands. These cells showed morphology compatible to macrophages with large nucleus and pale pink cytoplasm (**Figures 6A–D** amplified sections). Morphometric analyses revealed that, in the absence of SGE, larger numbers of L1-positive cells were observed in the L88-infected animals at weeks 8 and 10 PI compared to the Cobaia strain (**Figure 7A**). Interestingly, the presence of SGE modulated L1 presence in both strains, where their numbers were affected in all weeks PI (**Figure 7B**). In the absence of SGE, L88-infected animals showed a higher number of L1 positive cells in the 6th, 8th, and 10th weeks PI than those without SGE (**Figure 7C**). For Cobaia strain, only in the 12th week PI, the presence of SGE reduced the number of L1 positive cells (**Figure 7D**). Similar to L1, the presence of CD163 macrophages with expected morphology was also identified in lesions from both strains in the presence/absence of SGE (**Figures 8A–D** amplified sections). In the absence of SGE, no difference in the number of cells was detected by morphometry except in the 4th week (**Figures 8A,C, 9A**). In general, SGE influence was noticed during the 4th, 8th, and 10th weeks PI. A higher number of CD163+ cells was detected in L88-infected animals during the 4th and 8th weeks PI, whereas for Cobaia strain its number peaks at the 10th week PI (**Figures 8B,D, 9B**). While analyzing SGE presence according to strains, its effect was much more pronounced in the Cobaia strain than in L88 strain (**Figures 9C,D**). In Cobaia-infected animals, CD163+ cells were highly attracted during the 4th, 6th, 8th, 10th, and 12th week PI.

## Discussion

Several studies have addressed the role of *L. enriettii* as a model for CL (Bryceson et al., 1974; Belehu and Turk, 1976; Schottelius, 1987). However, none of those studies have used SGE in order to mimic natural infection. Here, this condition was included and in fact, its presence was able to modulate not only the initial but also the final steps of infection. More importantly, we demonstrated the role of L1 and CD163 macrophages in this process, an unknown aspect of the immunopathology caused by *Mundinia* species.

Similar to previous observations (Paranaíba et al., 2015), differences in pathogenicity of the strains were observed. In the presence/absence of SGE, L88 strain developed ulcerated/nodular lesions in *C. porcellus*, whereas Cobaia strain developed only non-ulcerated nodular lesions. An interesting aspect of this work is that in L88-infected animals, not all developed ulcerative lesions in the presence of SGE. However, some exhibited areas of hyperemia. This is consistent with an immunomodulation of the lesion by SGE. A few studies have addressed the histopathology in other members of the *Mundinia* subgenus. *Leishmania macropodum* can cause an opportunistic infection in the red kangaroos in Australia (Dougall et al., 2009). Under natural conditions, this infection presented a characteristic chronic proinflammatory infiltrate common to most *Leishmania* species (Marques et al., 2017). In the previous studies with *L. enriettii* from Brazil, this pattern was also observed during experimental conditions (Paraense, 1953; Bryceson et al., 1974; Ecco et al., 2000). Microscopically, those previous reports showed that a proinflammatory infiltrate with parasitized macrophages was very common. Some of the lesions could be ulcerated or not, with the presence of acanthosis and hyperkeratosis.

The role of saliva is very important during the inoculation of the parasite by the sand fly vector. In spite of having a wide range of pharmacologically active substances to help blood-feeding, the saliva of arthropod vectors also possesses substances that can act as immunomodulators for the parasites they transmit (Andrade et al., 2005). Monteiro et al. (2007) incriminated saliva from *L. longipalpis* infected with *L. major* as responsible for a higher intense leukocyte recruitment and Th2 cytokine modulation at the inoculation site. Laurenti et al. (2009) showed that SGE from field and laboratory-reared *L. longipalpis* differently affected the dermal lesions in mice experimentally infected with *L. amazonensis*. A discrete dermal lesion with lower parasite load and cellular inflammatory infiltration was noticed in those inoculated with SGE from laboratory-reared sand flies. Consistent with those observations, we also observed that SGE from laboratory-reared *L. longipalpis* was able to modulate the lesions in both strains. Our data showed that in the presence of SGE, the histopathological features were affected in both strains depending on the week and the marker used. Interestingly, we observed that even in after 6 weeks of infection, we could detect its effect. For this reason, since none of the previous histopathological studies with *L. enriettii* used SGE, they are somewhat incomplete. For example, the pro-inflammatory infiltrate (HE) caused by L88 was always more intense than that of Cobaia strain in the absence of SGE (**Figures 2A,B,D**) confirming its higher virulence (Paranaíba et al., 2015). In the presence of SGE, the pro-inflammatory infiltrate (HE) did not differ between L88 and Cobaia strain in the first month of infection (**Figure 2E**). Those data were confirmed by morphometry. While comparing each strain separately, the role of SGE did not affect the progression of L88 strain (**Figures 3A,B,E**). On the other hand, SGE exacerbated the pro-inflammatory infiltrate in Cobaia infection especially until the first month of infection (**Figures 3C,D,F**). Those data confirmed the immunomodulation of saliva together with the parasite for the differences observed in the progression of the lesions.

In order to better detect the amastigote forms of the parasite as well as the macrophages involved in the immunopathology of CL we performed IHC experiments. IHC parasite detection was successfully used to demonstrate the amastigote presence in the lesioned tissues in both strains. Again, L88 lesions were more stained than those originated from Cobaia strain also confirming their virulence (**Figures 4, 5**). The presence of SGE was able to exacerbate the parasite load in both strains until the 4th week PI. However, after the 6th week, in L88 strain lesions, the pro-inflammatory infiltrate took longer to heal than Cobaia strain (**Figure 5B**). Different from HE, IHC was able to detect the role of SGE in L88 infection especially in the 6th where it exacerbated the pro-inflammatory infiltrate (**Figure 5C**). These differences are expected since IHC is a more sensitive technique. Finally, in the presence of SGE, morphometric analysis of the less pathogenic Cobaia strain did not affect the course of lesion. However, it is interesting to notice that even in a low parasite load, this strain was able to cause inflammation that could be shown by HE after the 6th week (**Figure 2E**). Those data reinforce the use of more than one staining technique in order to better understand the immunopathological processes in *Leishmania*. However, the role of macrophages in this process have not been addressed in any studies with *L. enriettii*.

L1 subtype macrophages are usually present as immature and inflammatory cells at the site of the lesion and their differentiation is promoted by the myeloid-related protein (MRP) 14. The role of those cells was related to *L. major* infection susceptibility in mice (Steinbrink et al., 2000; Goto et al., 2007). Consistent with those observations, L1 macrophages were successfully detected in *L. enriettii*-infected animals. Following the peak of infection in the 6th week, the amount of L1 positive cells increased as demonstrated by IHC especially in the absence of SGE for L88 strain (**Figure 6A**). In general, in Cobaia strain, those differences were less noticed (**Figures 6B,D**). Remarkably, L1 positive cells were drastically decreased for both strains in the presence of SGE as demonstrated by morphometric analysis (**Figure 6B**). It is important to notice that in spite of appearing in the early events of infection, the peak of L1 positive cells coincides with the disappearance of lesion after the 6th PI especially in the L88-infected animals. This suggests their possible role in the control of the infection especially in the absence of SGE. Interestingly, in the presence of SGE, L1 positive cells were not observed in higher numbers, except for Cobaia strain in the 12th week PI. Those data show that SGE was able to modulate the presence of L1 positive cells throughout the course of infection. This reinforces that the saliva is important not only for the early events but also for the entire course of the infection helping parasites to circumvent some of the immunological mechanisms against them. It is important to mention that even in the presence of SGE, the lesions healed despite the presence of low number of L1 positive cells and perhaps this is more related to the strain. However, we do not know if SGE could promote visceralization in *L. enriettii*. It was recently reported (Dey et al., 2018) that vector-derived egested components were important for IL-1β and visceralization in *L. donovani*. Although they are completely different *Leishmania* species, visceralization of *L. enrieettii* was reported by Muniz and Medina (1948) after following the guinea pigs over a year.

Regarding CD163 positive cells, an opposite effect of the SGE compared to L1 cells was observed. CD163 macrophages are more mature macrophages as compared to L1. CD163 is a member of the scavenger receptor cysteine-rich family largely expressed on monocytes/macrophages and neutrophils presenting several roles as an extracellular sensor for bacteria and modulator of immunological responses (Fabriek et al., 2009; Moura et al., 2012). This receptor has been identified as an indicator of anti-inflammatory macrophages (M2 macrophages) (Kristiansen et al., 2001). Silva et al. (2017) demonstrated that in VL, the levels of soluble CD163 (sCD163) may serve as a biomarker for the severity of the disease in various inflammatory disorders. Its level was increased in VL patients and was directly correlated with clinical parameters of disease severity. Different from a viscerotropic species, this correlation was not detected in *L. enriettii*, since the less pathogenic strain exhibited higher CD163 expressing macrophages, and thus could be a source of sCD163. Those differences are probably related to the site of infection that varies dramatically between dermotropic and viscerotropic species. M2 macrophages have anti-inflammatory and tissue repair properties and are known to express CD163 (Li et al., 2015). With the exception of only one animal in the 4th week PI (**Figures 8A, 9A**), those cells were in very low numbers in the absence of SGE (**Figures 9A,C**). On the other hand, in the presence of SGE, those cells were remarkably attracted to the lesion site (**Figures 8D, 9B,D**). Those data suggest that the higher attraction of CD163 positive cells by Cobaia parasites may help to understand their less severe lesion (non-ulcerated) compared to L88 strain in the presence of SGE (**Figure 9B**). Also, those cells might be related to the better resolution of the infection in comparison to L88 strain. Altogether those data confirmed the role of those macrophages subtypes in the control/repair of lesions during the immunopathology of *L. enriettii*.

## Conclusion

It has been advocated for many years that *L. enriettii* could be a very useful model to study CL. Although this species shares many histopathological similarities with other dermothropic species, this statement should be revised. Both strains healed regardless of the presence/absence of SGE. However, the kinetics of this process varied completely, reinforcing the need of using SGE for other *Leishmania* species to better mimic infection. After molecular studies, *L. enriettii* has now been included in the subgenus *Mundinia*, whereas *L. braziliensis/L. guyanensis* and *L. amazonensis* belong to the Subgenus *Viannia* and *Leishmania*, respectively. An important feature of *L. enriettii* that differ this species from these dermothropic species is its ability to spontaneously heal after 12 weeks. For this reason, the data obtained in this work reinforces this model to understand mechanisms of control and repair in CL especially with the presence of subtypes of macrophages affecting this phenomenon in the presence of SGE.

## Author Contributions

LJP, LFP, and PP performed the experiments. AA performed the data analysis. LJP, NG, RS, and WT did the data analysis and wrote the manuscript. LJP and LFP contributed equally to the manuscript.

## Conflict of Interest Statement

The authors declare that the research was conducted in the absence of any commercial or financial relationships that could be construed as a potential conflict of interest.

## Supplementary Material

FIGURE S1 Negative controls of histological (HE) and immunohistochemistry (IHC) of nasal areas of *C. porcellus.* **(A)** Inoculated with saline, **(B)** inoculated with SGE, **(C)** uninfected animal, **(D)** inoculated with saline (negative control for amastigote labeling), and **(E)** inoculated with saline (negative control for L1 and CD163 labeling).
