# Supplementary figures and images for: Salivary Gland Extract Modulates the Infection of Two Leishmania enriettii Strains by Interfering With Macrophage Differentiation in the Model of Cavia porcellus
Source: Front Microbiol. 2018 May 29;9:969. doi: 10.3389/fmicb.2018.00969 (PMC5986888; doi:10.3389/fmicb.2018.00969)

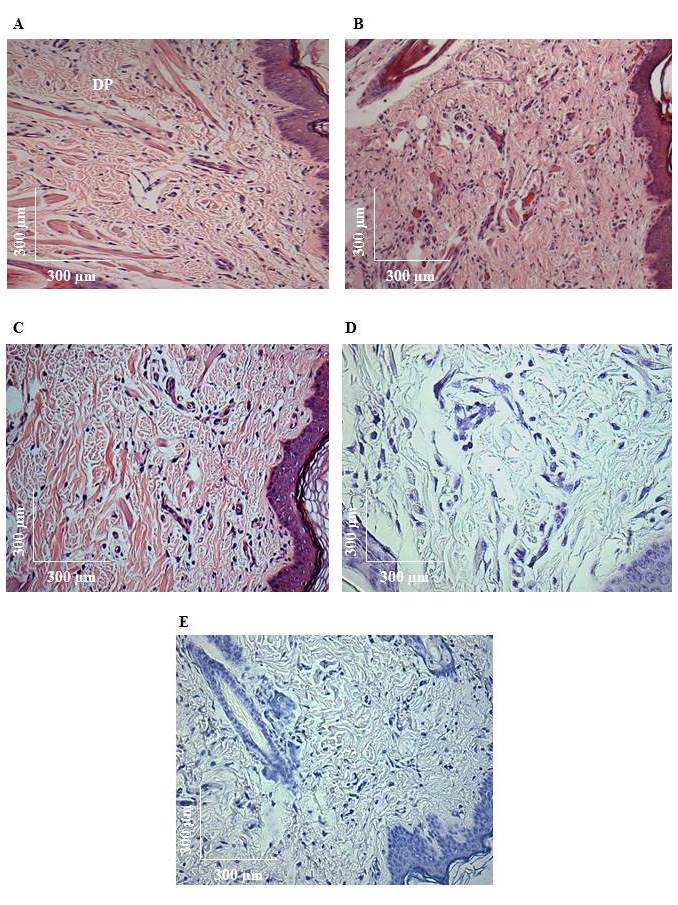

Supplement: FIGURE S1 — Negative controls of histological (HE) and immunohistochemistry (IHC) of nasal areas of C. porcellus. (A) Inoculated with saline, (B) inoculated with SGE, (C) uninfected animal, (D) inoculated with saline (negative control for amastigote labeling), and (E) inoculated with saline (negative control for L1 and CD163 labeling). [file Image_1.jpeg]
